# Cenobamate modulates EEG cortical activity and connectivity in individuals with drug-resistant epilepsy: a pharmaco-EEG study

**DOI:** 10.3389/fneur.2024.1502668

**Published:** 2025-03-03

**Authors:** G. Assenza, B. Sancetta, L. Ricci, C. Vico, F. Narducci, M. Boscarino, J. Lanzone, P. Menna, C. Liguori, F. Izzi, N. B. Mercuri, V. Di Lazzaro, M. Tombini

**Affiliations:** ^1^Research Unit of Neurology, Department of Medicine and Surgery, Università Campus Bio-Medico di Roma, Rome, Italy; ^2^Operative Research Unit of Neurology, Fondazione Policlinico Universitario Campus Bio-Medico, Rome, Italy; ^3^Neurorehabilitation Department of the Milano Institute, Istituti Clinici Scientifici Maugeri IRCCS, Milan, Italy; ^4^Operative Research Unit of Clinical Pharmacology, Fondazione Policlinico Universitario Campus Bio-Medico, Rome, Italy; ^5^Neurology Unit, Sleep Medicine Centre, University Hospital of Rome Tor Vergata, Rome, Italy

**Keywords:** cenobamate, pharmaco-EEG, EEG connectivity, drug-resistant epilepsy, response biomarker

## Abstract

**Objective:**

Quantitative electroencephalography (qEEG) metrics are demonstrated to correlate with and predict clinical response in individuals with epilepsy. Cenobamate is an effective anti-seizure medication recently approved as an add-on therapy for individuals with epilepsy, but its effects on qEEG are unknown. We aimed to evaluate the modulation of qEEG metrics induced by cenobamate and its relationship with clinical response.

**Methods:**

We performed a prospective study with a cohort of 18 individuals with epilepsy (8 women, 47 ± 16 years old) and 25 healthy subjects (HS). They underwent a 19-channel EEG before and 6 months after cenobamate administration. Power spectral density (PSD) and phase locking value (PLV) for delta, theta, alpha, beta, and gamma frequency bands were calculated. Correlation analysis and analysis of covariance exhibited significant cenobamate-induced changes in qEEG and their relationship with seizure frequency changes. A regression analysis was performed to evaluate the association with clinical responders.

**Results:**

A total of 11 out of 16 individuals with epilepsy (69%, with 2 dropping out) were cenobamate responders (≥50% seizure frequency reduction). Cenobamate did not modify any PSD parameter but induced significant changes in PLV levels (*p* < 0.01). A decrease in PLV correlated with seizure reduction (*p* < 0.03). Regression analysis showed a strong association between PLV modulation and cenobamate responsiveness (a sensitivity of 0.75, a specificity of 0.84, and an accuracy of 0.81).

**Conclusion:**

Cenobamate induces an EEG connectivity modulation that is highly associated with cenobamate clinical response.

**Significance:**

Connectivity analysis of pharmaco-EEG can provide new hints toward the development of innovative biomarkers and precision medicine in individuals with epilepsy.

## 1 Introduction

Epilepsy is a chronic neurological disorder characterized by an enduring predisposition to generate seizures (1). One-third of individuals with epilepsy suffer from drug-resistant epilepsy, a condition defined as the persistence of seizures despite appropriate medications (2). Cenobamate is a new anti-seizure medication (ASM) recently approved by the US Food and Drug Administration and the European Medicines Agency for the treatment of focal-onset seizures in adults with drug-resistant epilepsy (3). Cenobamate has been shown to enhance GABA-A receptor-mediated inhibitory currents (4) and block excitatory currents by promoting the inactivated state of voltage-gated sodium channels, although its exact mechanism of action is unknown (5).

A growing body of evidence suggests that different ASMs can influence cortical activity and its excitability (6–8). Electroencephalogram (EEG) provides information on mesoscale spontaneous neural cortical activity, making it an ideal non-invasive technique to study neural network disorders such as epilepsy (9, 10). The interpretation of epilepsy as a network disorder is based on the clinical evidence that epileptic activity involves and impairs the functioning of cortices sometimes remote and distant from the epileptic focus (11–13). Quantitative EEG (qEEG) analysis is a promising tool for investigating epileptic network pathophysiology. The branch of research that investigates the qEEG effect of specific drugs on electric brain activity through qEEG analysis is known as pharmaco-EEG (14). In the field of epilepsy, pharmaco-EEG studies may have a multi-level potential. Indeed, our previous studies exploring scalp EEG connectivity sensitive to clinical response reported that different ASMs may induce functional EEG connectivity changes that parallel clinical response; moreover, our preliminary data also show that such modulation can predict the response to the first ASM in newly diagnosed individuals with epilepsy (6, 8, 15, 16).

Along this line, with the present study, we aimed to test (i) the cenobamate effect on the EEG cortical activity and connectivity using qEEG analysis and (ii) whether cenobamate-induced qEEG modulation correlates with clinical response. To reach this goal, we (i) retrospectively collected a cohort of individuals with drug-resistant epilepsy undergoing add-on therapy with cenobamate; (ii) compared the qEEG metrics before and 6 months after cenobamate administration, alongside a cohort of healthy subjects (HS); (iii) performed correlation analysis between the qEEG metrics and seizure reduction after cenobamate administration; (iv) performed logistic regression analysis to test the ability of the qEEG metrics to distinguish cenobamate responders (>50% seizure frequency reduction) from cenobamate non-responders.

## 2 Materials and methods

### 2.1 Participants’ recruitment and clinical evaluation

**Figure 1:**
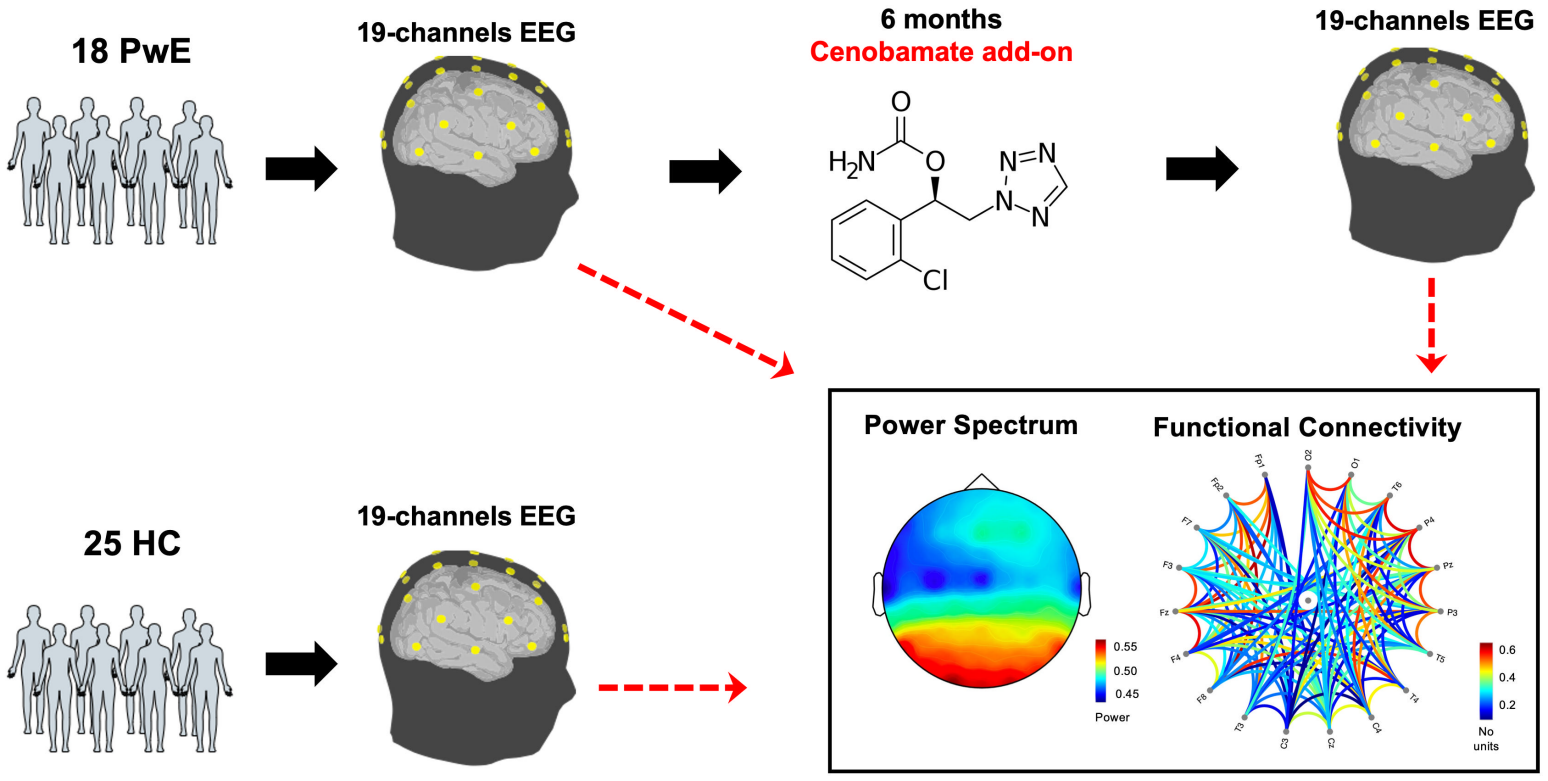
Study design. PwE, people with epilepsy; EEG, electroencephalogram; HS, healthy subjects; T0, before cenobamate administration; T1, 6 months after cenobamate administration.

We conducted a prospective, longitudinal, multi-center study. We enrolled a group of individuals with focal-onset drug-resistant epilepsy, eligible for add-on therapy with cenobamate, who attended the outpatient clinics of the epilepsy centers of the Campus Bio-Medico University Hospital Foundation of Rome and the University Hospital Tor Vergata of Rome from January 2021 to February 2022, as well as a cohort of healthy subjects (HS) who were matched for age and sex. The study design is shown in Figure 1.

The inclusion criteria for the individuals with epilepsy group were as follows: (i) diagnosis of focal epilepsy supported by the International League Against Epilepsy’s (ILAE) working definition (17); (ii) drug-resistant epilepsy diagnosis according to the current ILAE definition, i.e., failure of at least two tolerated ASMs with appropriate and tolerated therapy and dosage (18); (iii) individuals with epilepsy who underwent at least one imaging examination in the previous 5 years to have a complete clinical characterization; and (iv) individuals with epilepsy meeting the Italian-approved indication for prescribing cenobamate (adjunctive therapy of focal-onset epileptic seizures with or without secondary generalization in adult patients with epilepsy who have not achieved adequate seizure control despite prior treatment with at least two anti-epileptic drugs) and planned to start cenobamate as add-on therapy.

The exclusion criteria for the individuals with epilepsy group were as follows: (i) individuals with epilepsy who could not comply with EEG recording; (ii) EEG quality that was deemed not sufficient; (iii) individuals with epilepsy who were under medications known to affect EEG, other than ASMs (i.e., antidepressants); (iv) individuals with epilepsy affected by any significant neurological condition other than epilepsy (i.e., previous stroke or brain tumor); and (v) excessive sleepiness before EEG recording, as tested immediately before EEG recording by a Stanford Sleepiness Scale score > 3 (19) and/or EEG signs of drowsiness or sleep.

The inclusion criteria for the HS group were as follows: (i) subjects who could comply with EEG recording; (ii) EEG quality that was deemed sufficient; (iii) subjects who were not under medications known to affect the EEG (i.e., antidepressants); (iv) subjects who were not affected by any known neurological disease; (v) no pathological findings at the neurological examination; and (vi) excessive sleepiness before EEG recording, as tested by a Stanford Sleepiness Scale score > 3.

Individuals with epilepsy underwent EEG recording <7 days before administering cenobamate (T0) and 6 months after cenobamate initiation (T1). No changes in ASM other than cenobamate were allowed from T0 to T1. If ASM changes were clinically required, then the subjects were excluded from the subsequent analyses.

The final cenobamate dose was tailored for each patient depending on tolerance and clinical response (minimal effective dose or maximal tolerated dose). Titration was managed with dosage increases every 2 weeks aiming to achieve a minimal dosage of 200 mg/day. If adverse effects were reported, the titration was slowed, or the final dosage was reduced (20). A basal EEG was recorded in HS to obtain normative data. Written informed consent was obtained from all participants. All procedures were in accordance with the ethical standards of the 1964 Declaration of Helsinki and its later amendments. Additionally, individuals with epilepsy were asked to closely monitor and report adverse effects related to cenobamate and to keep a detailed seizure diary from 3 months before starting cenobamate until T1. The seizure frequency (SF) was considered the monthly mean number of seizures of the last 3 months before administering cenobamate (SF_T0_) and 6 months after cenobamate administration T1 (SF_T1_). For those individuals with epilepsy with significant cognitive impairment, caregivers provided the same clinical data.

### 2.2 EEG recordings and analysis

All participants underwent a 10 min 19-channel EEG recording, with standard 10–20 montage according to the IFCN guidelines (21). In accordance with the International Pharmaco-EEG Society, recordings were performed in the morning (between 9 and 11 a.m.) during quiet rest with eyes open in a sound-attenuated room, with constant dimmed light, and in a reclined comfortable position using a 32-channel Micromed system (SystemPlus software; Micromed, Mogliano Veneto, Italy). The day before the recording, it was recommended to avoid alcohol consumption for 24 h and to avoid caffeine and tobacco smoking the morning before the recording (22).

The EEG preprocessing and qEEG analysis were performed using Brainstorm Toolbox for Matlab (Math Works Inc., Natick, MA) (23). EEGs were preprocessed with the following pipeline: (i) EEG re-reference to common average reference; (ii) 49–51 Hz notch filter; (iii) band-pass filter between 0.5 and 70 Hz; (iv) manual rejection of interictal epileptiform abnormalities by an experienced neurophysiologist (LR, GA, and MT); and (v) correction for pulse, eye-blink, and muscular artifacts using Independent Component Analysis procedure supported by automatic component labeling (IClabel) (24). Each component labeled as an artifact by IClabel was visually checked (qualitative spectral and topography features) before rejection (25, 26). After preprocessing, from the original row signals, we selected 180 s of continuous epochs for further analysis (27).

To assess the effect of cenobamate on brain networks, we performed measures of resting-state brain activity and connectivity (27). As a measure of global cortical activity, we computed the average relative power spectrum density (PSD) over all channels using a standard fast Fourier transform approach (Welch’s method: average of non-overlapped windows with a duration of 2 s) for the following frequency bands: (i) delta: 2–4 Hz; (ii) theta: 5–7 Hz; (iii) alpha: 8–12 Hz; (iv) beta: 13–29 Hz; and (vi) gamma: 30–60 Hz (6). As a connectivity parameter, we focused on phase locking value (PLV), a measure of non-directional frequency-specific synchronization reflecting long-range integrations that assess the extent to which the phase difference between two signals changes over time (28, 29). We focused on the same frequency bands as for the power spectrum. The PLV levels were measured for all possible channel combinations and then averaged to obtain a measure of global connectivity (8).

### 2.3 Statistical analysis

The statistical analysis was performed using the JASP (Ver. 0.16.4, Apple silicon, University of Amsterdam, Netherlands) and Matlab (MathWorks) software.

#### 2.3.1 Descriptive statistics and grouping

Normality distribution was checked using the Shapiro–Wilk test. Normally distributed variables (*p*-value of Shapiro–Wilk >0.05) were reported as mean ± standard deviation, while non-normally distributed variables were reported as median and interquartile ranges (IQR). The mean age of HS and individuals with epilepsy was compared using a two-tailed *t*-test. Individuals with epilepsy were grouped into cenobamate responders (seizure reduction ≥50% at T1 with respect to T0) (18) and cenobamate non-responders.

#### 2.3.2 Inferential statistics

##### 2.3.2.1 Correlation analysis

The following EEG variables were selected for correlation analysis: PSD and PLV levels at T0 (PSD_T0_ and PLV_T0_, respectively), PSD and PLV levels at T1 (PSD_T1_ and PLV_T1_, respectively), percentage variation of PSD levels induced by cenobamate (PSD_T1−T0_, i.e., (PSD_T1_–PSD_T0_)/PSD_T0_), and percentage variation of PLV levels induced by cenobamate (PLV_T1−T0_, i.e., (PLV_T1_–PLV_T0_)/PLV_T0_). Spearman’s correlation analysis was performed among EEG variables, percentage of seizure frequency variation induced by cenobamate [SF_T0−T1_, i.e., (seizure frequency at T0 – seizure frequency at T1)/seizure frequency at T0], epilepsy duration, number of ASMs, and baseline seizure frequency.

##### 2.3.2.2 Comparison between individuals with epilepsy and HS

We applied a mixed-model repeated measures analysis of variance (ANOVA), with *Band* (delta, theta, alpha, beta, and gamma) as a within-subjects factor and *Group* (two levels: HS and individuals with epilepsy at T0; HS and individuals with epilepsy at T1) as a between-subjects factor to compare the individuals with epilepsy and HS groups. Mixed ANOVA was conducted twice, for both PSD and PLV levels. The Greenhouse–Geisser correction was applied when needed.

##### 2.3.2.3 Comparison between T0 and T1 in individuals with epilepsy

The effect of cenobamate on PSD and PLV levels within the individuals with epilepsy group was tested with a two-way repeated measures analysis of covariance (ANCOVA), with *Band* (five levels: delta, theta, alpha, beta, and gamma) and *Time* (two levels: T0 and T1) as within-subjects factors; as *covariates*, we selected all variables moderately or strongly correlated with PLV_T1−T0_ and/or PSD_T1−T0_. Mauchly’s test was used to evaluate the sphericity assumption, and the degrees of freedom were corrected using the Greenhouse–Geisser procedure.

The significance level was set at *p* < 0.05. The Benjamini–Hochberg false discovery rate procedure was used for *post-hoc* correction for multiple comparisons when needed.

##### 2.3.2.4 Logistic regression

Logistic regression was performed to test the ability of qEEG variables to classify our cohort of individuals with epilepsy according to cenobamate clinical response (cenobamate responders vs. cenobamate non-responders). The model was tuned with the following EEG variables, for each frequency band: PSD_T0_, PLV_T0_, PSD_T1_, PLV_T1_, the magnitude of PLV modulation induced by cenobamate (PLV_T0−T1_, i.e., (PLV_T0_–PLV_T1_)/PLV_T0_), and the magnitude of PSD modulation induced by cenobamate (PSD_T0−T1_, i.e., (PSD_T0_–PSD_T1_)/PSD_T0_). The model was imputed with PLV_T0−T1_ and PSD_T0−T1_ (instead of PLV_T1−T0_ and PSD_T1−T0_, used for correlation analysis) to facilitate results interpretation.

Regularization with the least absolute shrinkage and selection operator with L1 penalty was applied to prevent the algorithm from overfitting the dataset. The model was tuned with all EEG variables. For each feature imputed in the model, we reported the associated linear coefficient (βs) to express feature salience in discriminating cenobamate responders. To assess the performance of the model, we built the receiver operating characteristic (ROC) curve and confusion matrix; sensitivity (Sens), specificity (Spec), positive predictive value (PPV), negative predictive value (NPV), accuracy (Acc), and ROC curve’s area under the curve (AUC) were computed.

## 3 Results

### 3.1 Descriptive statistics and grouping

We enrolled a group of 18 drug-resistant individuals with epilepsy (female/male ratio: 8/10, age: 46 ± 16 years old) and a group of 25 HS (female/male ratio: 13/12, age: 51 ± 18 years old) who met all inclusion and exclusion criteria of the study. There was no significant difference in mean age between the individuals with epilepsy and HS groups. Demographic characteristics and clinical variables of individuals with epilepsy are presented in Table 1. Two individuals with epilepsy dropped out of the study (one due to increased seizure frequency and the other due to poor medical adherence) and were not included in the follow-up.

**Table 1:** Clinical variables of our cohort in aggregated form.

Variables	*N*	Mean	SD	Range
Groups				
PwE	18			
HS	25			
Gender (F/M)				
PwE	8/10			
HS	12/13			
Age (years)				
PwE		47	16	25–75
HS		51	18	21–70
Age of epilepsy onset (years)		12.8	14.7	1–49
Epilepsy duration (years)		34	15.9	5–57
Epilepsy etiology				
Structural	10			
Genetic	2			
Unknown	6			
Surgery	0			
VNS	11			
Psychiatric comorbidity	7			
Cognitive disability	8			
Anti-seizure medication (*N*)		3	1	2–7
CNB dosage at T1 (mg/day)		171.9	70.6	
Seizure *N* at T0 *(monthly)*		11.5	11.2	
Seizure *N* at T1 *(monthly)*		5	7.5	
Drop-out	2 PwE with VNS			
Responders at T1	11			
Seizure free at T1	2			

PwE, people with epilepsy; HS, healthy subjects; *N*, number; VNS, vagal nerve stimulation; SD, standard deviation; T0, before CNB introduction; T1, after 6 months from T0.

In the individuals with epilepsy group, the mean age of epilepsy onset was 12.8 ± 14.7 years old, the mean epilepsy duration was 34 ± 15.9 years old, and the median number of ASMs was 3 (IQR:1, range: 2–7). In total, 11 out of 16 individuals with epilepsy (69%) had vagal nerve stimulation, and among them, 6 individuals with epilepsy (55%) were vagal nerve stimulation responders. No individuals with epilepsy underwent epilepsy surgery. At T0, individuals with epilepsy had a median number of seizures (monthly mean of the last 3 months) equal to 11.5 ± 11.2. At T1, 11 out of 16 individuals with epilepsy (69%) were cenobamate responders, and among them, 2 individuals with epilepsy achieved complete seizure freedom (12%). The median number of seizures (monthly mean of the last 3 months) at T1 was equal to 5 ± 7.5. The median reduction in seizure frequency was 55% (IQR 58.1, range 0–100). During the titration of the cenobamate therapy, 12 individuals with epilepsy (70%) reported non-serious adverse events (epilepsy ataxia in 5 individuals, epilepsy somnolence in 5 individuals, headache in 1 subject, and asthenia in 1 subject). These adverse events were mitigated by slower titration or a cenobamate daily dose adjustment.

### 3.2 Inferential statistics

#### 3.2.1 Correlation analysis

**Figure 2:**
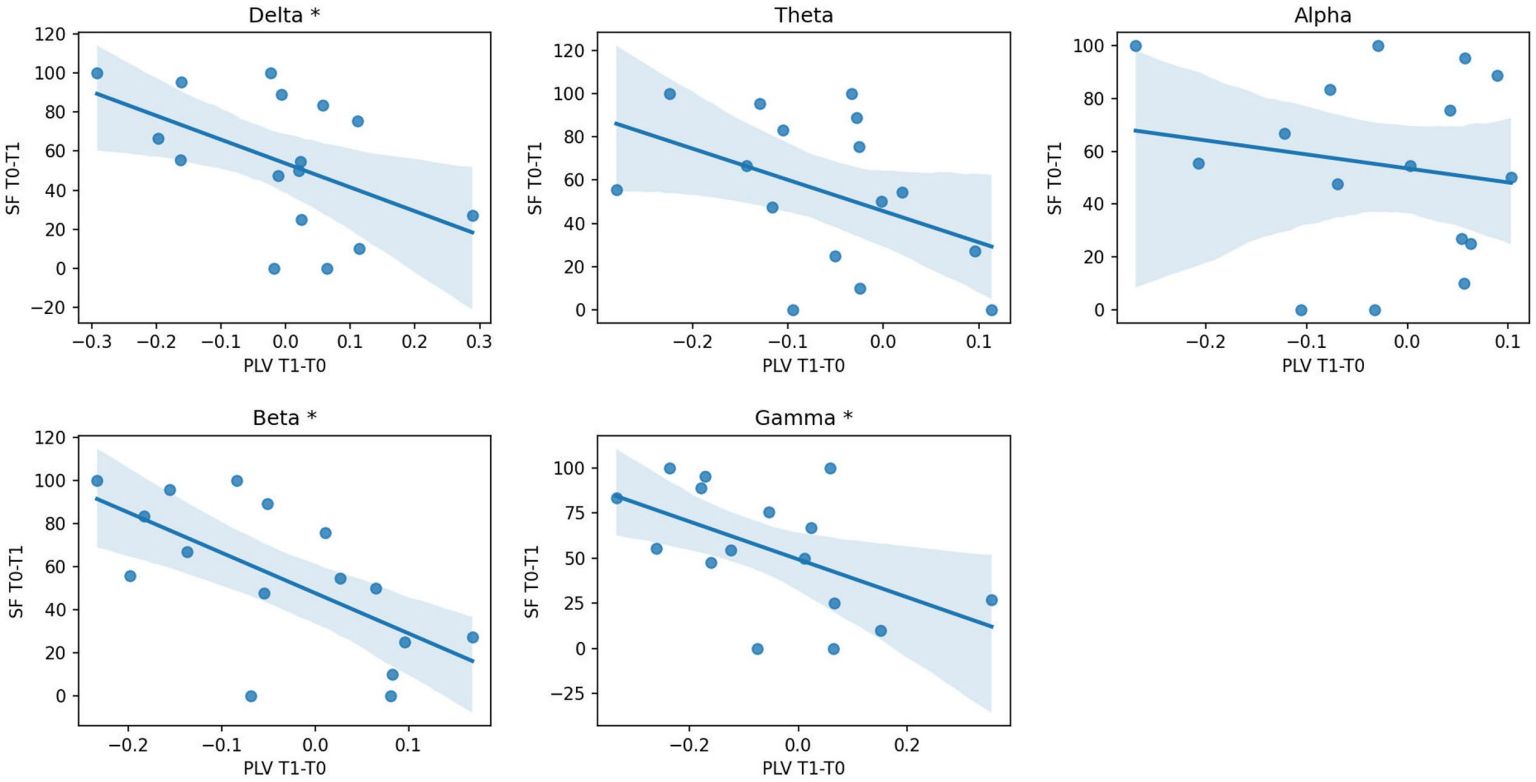
Correlation analysis between EEG PLV changes and seizure response after CNB. Scatterplot and regression line of seizure reduction percentage (SF_T0−T1_, dependent variable) over percentage variation of PLV connectivity T1 vs. T0 (PLV_T1−T0_, independent variable) for each frequency band. * *p*-value of correlation coefficient < 0.05; PLV, phase locking value; PLV_T1−T0_, percentage variation of PLV connectivity T1 vs. T0; SF_T0−T1_, percentage variation of seizure frequency T1 vs. T0; T0, before CNB administration; T1, 6 months after CNB administration.

SF_T0−T1_ was inversely correlated with PLV_T1−T0_ in all frequency bands (delta: rho −0.52, *p* 0.04; beta: rho −0.68, *p* 0.004; gamma: rho −0.53, *p* 0.04) except for alpha and theta (as illustrated in Figure 2), showing that a reduction of PLV induced by cenobamate was associated with a better clinical response to cenobamate. PLV_T1−T0_ of the theta band showed a good trend of correlation, but it did not reach significance (rho 0.46, *p* 0.07). SF_T0−T1_ did not statistically correlate with any frequency band of PSD_T1−T0_. PSD_T0_ and PSD_T1_ did not show any significant correlation with clinical variables.

Epilepsy duration and the number of ASMs were significantly and inversely correlated with gamma PLV_T0_ (rho −0.56; *p* 0.03). Age of epilepsy onset and baseline seizure frequency did not show any significant correlation with any EEG variable.

#### 3.2.2 Comparison between individuals with epilepsy and HS

The mixed-model ANOVA revealed a significant Band*Group interaction for both PSD_T0_ and PLV_T0_ (F statistic 59.82, *p*-values <0.001). The *post-hoc* analysis showed that the individuals with epilepsy group had significantly lower PSD_T0_ for fast frequencies (alpha, beta, and gamma), higher PSD_T0_ for slow frequencies (delta and theta), and lower PLV_T0_ for the alpha frequency band than those of the HS group (*p*-values <0.001).

ANOVA also revealed a significant Band*Group interaction for both PSD_T1_ and PLV_T1_ (F statistic 44.14, *p*-values <0.001). The *post-hoc* analysis revealed that the individuals with epilepsy group had significantly lower PSD_T1_ for fast frequencies and higher PSD_T1_ for slow frequencies than those of the HS group (*p*-values <0.001). Additionally, individuals with epilepsy exhibited lower PLV_T1_ across all frequency bands than those of the HS group (*p*-values between 0.01 and < 0.006), except for the delta frequency band.

#### 3.2.3 Comparison between T0 and T1 in individuals with epilepsy

**Figure 3:**
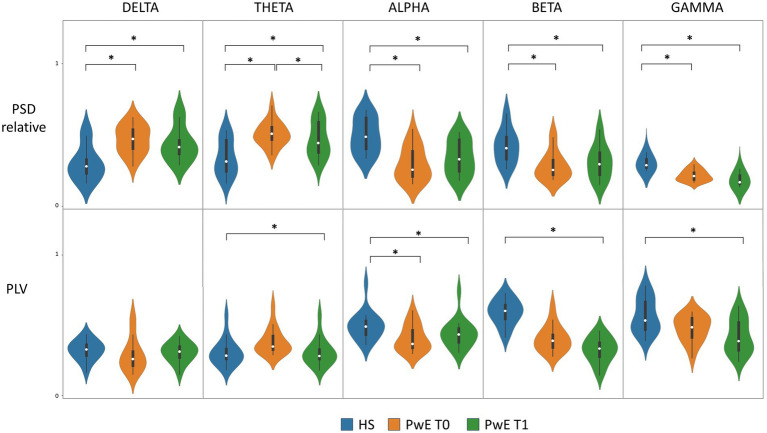
PSD and PLV levels in PwE and HS. Boxplot and violin plot of PLV and relative PSD distribution for HS, PwE at T0, and PwE at T1 for each frequency band. CNB induced a significant overall PLV modulation between T0 and T1 as shown by ANCOVA with SF_T0−T1_ as *covariate* (not shown in the figure). * *post-hoc* analysis of ANOVA/ANCOVA models showing a *p*-value <0.05; ANCOVA, analysis of covariance; HS, healthy subjects; PLV, phase locking value; PSD, power spectral density; PwE, people with epilepsy; T0, before CNB administration; T1, 6 months after CNB administration.

The results of repeated measures ANOVA and ANCOVA (individuals with epilepsy at T0 vs. individuals with epilepsy at T1) are shown in Figure 3. The two-way repeated measures ANOVA revealed a significant influence of the within-subjects factor *Time* on PSD values (F statistic 4.63, *p*-value 0.05) as well as the existence of the significant Time*Frequency interaction (F statistic 4.78, *p*-value 0.03). The *post-hoc* analysis showed that the individuals with epilepsy group had significantly lower PSD_T1_ for the theta frequency band than that of PSD_T0_ (*p*-value 0.01).

Because of the existence of a significant moderate correlation between SF_T0−T1_ and PLV_T1−T0_ (as shown by correlation analysis), we applied a two-way repeated measures ANCOVA on PLV with SF_T0−T1_ as the *covariate*. ANCOVA showed a significant influence of the within-subjects factor *Time* on PLV distribution (F statistic 6.48, *p*-value 0.01) and a significant Time*Band interaction (F statistic 4.32, *p*-values 0.03), thus proving a statistically significant modulation on PLV levels induced by cenobamate in individuals with epilepsy. The *post-hoc* analysis failed to demonstrate the existence of a statistically significant difference for a specific frequency band.

#### 3.2.4 Logistic regression

**Figure 4:**
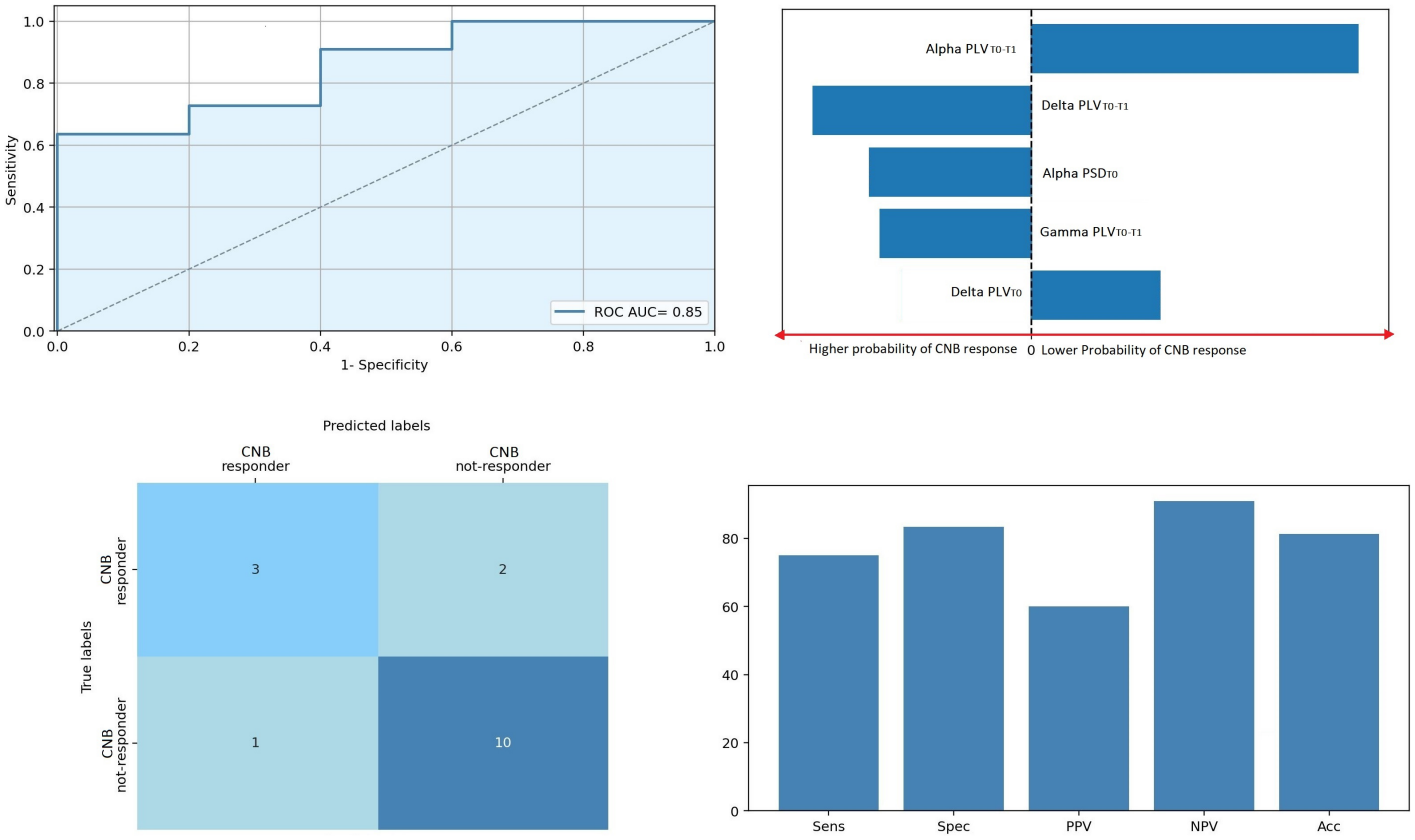
Regression model predicting clinical responsiveness. ROC curve of the regression model. Top right panel: Five most relevant features of the model; bar lengths are proportional to the magnitude of linear coefficients; predictors on the left side of the dotted line predicted a higher probability of clinical CNB response, the contrary for predictors on the right side of the dotted line. Bottom left panel: Confusion matrix of the regression model. Bottom right panel: Predictive performance of the predictive model. Acc, accuracy; CNB, cenobamate; NPV, negative predictive value; PLV, phase locking value; PLV_T0_, PLV levels at T0; PLV_T1−T0_, percentage variation of PLV connectivity T0 vs. T1; PPV, positive predictive value; PSD, power spectral density; PSD_T0_, PSD levels at T0; ROC AUC, area under the receiver operating characteristic curve.

The results of multiple logistic regression are shown in Figure 4. Logistic regression showed that EEG variables correctly discriminate individuals with epilepsy according to cenobamate clinical response with an ROC curve’s AUC = 0.85 (*p* < 0.001). Performances of the model were the following: Sens: 0.75, Spec: 0.84, PPV: 0.6, NPV: 0.91, and Acc: 0.81. Alpha, delta, and gamma PLV_T0−T1_, together with alpha PSD_T0_ and delta PLV_T0_, offered the highest influence on decision scores. Specifically, higher alpha PSD_T0_ is associated with a higher probability of clinical response to cenobamate (*β* −2.9), while higher delta PLV_T0_ is associated with a lower probability of response to cenobamate (*β* 2.3). Higher alpha PLV_T0−T1_ (higher difference between T0 and T1, i.e., higher reduction of PLV level induced by cenobamate in the alpha band) is associated with a lower probability of clinical response to cenobamate (*β* 6.8), while the higher magnitude of delta and gamma PLV_T0−T1_ (higher reduction of PLV level in delta and theta bands) is associated with a higher probability of clinical response to cenobamate (*β* −3.9 and *β* −2.7, respectively).

## 4 Discussion

In the present study, we evaluated the effects of add-on therapy with cenobamate on EEG cortical activity and connectivity in a cohort of individuals with epilepsy, comparing such results with a normative group of HS. We also assessed the correlation between cenobamate-induced changes in qEEG metrics and seizure frequency reduction. Finally, we tested the ability of cenobamate-induced spectral and connectivity modulation in discriminating our cohort according to cenobamate clinical response. Our main findings can be summarized as follows: (i) individuals with epilepsy had lower PSD for fast frequencies and higher PSD for slow frequencies than those of HS, both at T0 and T1; (ii) cenobamate treatment induces an overall significant modulation of both PSD and PLV levels; (iii) the magnitude of PLV reduction significantly correlates with seizure frequency response induced by cenobamate; and (iv) the modulation of PLV EEG connectivity associates with the variability of clinical response to cenobamate with a high level of sensitivity.

### 4.1 PSD results

Our study revealed that individuals with epilepsy exhibited significantly lower power in alpha, beta, and gamma frequency bands and higher power in delta and theta frequency bands than those of HS, both at T0 and T1. These results confirm the peculiar spectral phenotype observed in already pharmacologically treated individuals with epilepsy, which is characterized by an increase in slow frequencies and a decrease in high-frequency band power (30, 31). This spectral shift toward the slowest frequencies, more pronounced in the affected hemisphere of individuals with epilepsy (14, 31), could be related to pharmacological burden (31). Alternatively, it could mirror the epileptic activity itself (32, 33). Recent evidence suggests that slow EEG activity may reflect the ongoing epileptogenic process and the network alterations involving the neocortex rather than simply lesional activity or ASMs’ effect (32, 33), so much that the delta band is considered by some authors a scalp EEG biomarker of the epileptic network (34). In this perspective, we might speculate that the increased power in low-frequency bands in individuals with epilepsy could reflect the aberrant plasticity and long-term potentiation mechanisms induced by the epileptic network. However, without invasive recording, we cannot rule out that part of slow wave activity is an effect of deep epileptic focus or of ASM burden (35, 36).

Past pharmaco-EEG studies demonstrated a general reduction of spectral power induced by old-generation ASMs (such as valproate, gabapentin, and carbamazepine) (37–39). In contrast, recent pharmaco-EEG findings on new-generation ASMs showed non-univocal results. The variability of results could be attributed to the extreme heterogeneity of the sample groups in terms of epilepsy and seizure types, disease duration, lobar involvement, ratio of responders, and the number and type of assumed ASMs. In a previous study on the effects of eslicarbazepine acetate in individuals with epilepsy, our group did not report significant spectral changes, while we demonstrated an ASM-induced increase of theta and alpha power in the case of perampanel (6). In drug-naïve individuals with temporal lobe epilepsy, we previously demonstrated an increased EEG power for alpha and decreased power for theta after a treatment regimen with levetiracetam, with a return to values similar to HS. In the case of cenobamate, the present study revealed a significant reduction of PSD levels in the theta frequency band. Recurrent seizures are responsible for maladaptive network remodeling and long-term potentiation-like phenomena (40), which lead to an increase in slow-wave activity (31). In this perspective, we might speculate that theta power reduction may reflect the potential of cenobamate in disrupting the epileptic network activity underlying seizure onset and propagation, which is in line with the clinical efficacy demonstrated by cenobamate (41).

### 4.2 PLV results

Neurophysiological modeling studies from invasive recordings have unequivocally demonstrated that the brain tissue involved in the seizure onset and propagation can be divided into multiple networks, which are reciprocally connected with a strength proportional to their involvement in the seizure (42). In this context, functional connectivity is an essential measure of pathophysiological processes subtending epileptic network organization, cortical excitability, and seizure recurrence. EEG is an ideal tool for investigating functional connectivity as it can monitor both excitability and connectivity of the cortex with high temporal resolution (43).

In previous studies, we reported that treatment with eslicarbazepine acetate can induce EEG connectivity changes in individuals with focal epilepsy (6), while Lanzone et al. (8) and Routley et al. (7) reported different connectivity responses after chronic and acute administration of perampanel, respectively. In the case of levetiracetam, we observed a decline in EEG connectivity of the epileptic network, which provided the best classification results in terms of predicting seizure freedom after 2 years of a single ASM therapy (16, 44). Studies of non-pharmacological treatments also reported that EEG connectivity reduction paralleled a good clinical response (45, 46).

In the case of cenobamate, our study showed that cenobamate administration induces an overall change in PLV levels, although *post-hoc* analysis failed to detect a frequency-specific modulation effect (maybe due to the small sample size of our cohort of study). The magnitude of cenobamate-induced PLV modulation paralleled clinical outcomes (the higher the reduction of PLV level after cenobamate administration, the higher the seizure frequency reduction induced by cenobamate). Clinical amelioration after cenobamate administration was also associated with EEG connectivity modulation as shown by regression analysis. Specifically, the reduction of EEG connectivity associated with improved seizure frequency was observed across the entire EEG spectrum, except for the alpha frequency band. Moreover, the rate of reduction of connectivity within the alpha frequency band was strongly associated with a poor outcome in cenobamate response, in contrast to the findings for the other frequency bands. Alpha is the predominant physiological frequency in awake humans. It is correlated with attentional and cognitive tasks in healthy individuals (47, 48), and its reduction is observed in various neurological disorders, particularly those involving cognitive disturbances (49). Therefore, the distinct pattern of EEG connectivity modulation observed in our study, namely, the preservation of alpha frequency connectivity, may suggest that the reduction in EEG connectivity could be associated with pathological (epileptic) network activity.

As a standard of care of everyday epileptological clinical practice, dose adjustment of concomitant sodium channel blockers (daily dose reduction) during the administration of cenobamate is required to avoid treatment-emergent adverse effects (50). Moreover, sodium channel blockers are the principal ASMs administered for the treatment of focal-onset epilepsy (51). For this reason, it is impossible to evaluate the isolated impact of cenobamate administration. In our cohort, concomitant sodium channel blockers’ daily dose reduction was required only in individuals with epilepsy with a daily dose of cenobamate >100 mg/day. The concomitant sodium channel blockers were lacosamide, lamotrigine, eslicarbazepine acetate, and carbamazepine (as reported in Table 2). As demonstrated by previous pharmaco-EEG studies, lamotrigine and lacosamide have a poor influence on frequency-specific background EEG activities (52–55). Eslicarbazepine acetate has been shown to induce broad-band modulation in EEG cortical activity and connectivity, while carbamazepine has been proven to increase spectral activity in low frequencies (6, 37). However, these two ASMs were only administered to three individuals with epilepsy, each with a concomitant cenobamate daily dose >100 mg/day. For these motivations, background EEG modulation is not likely to be explained by the reduction of concomitant sodium channel blockers. Nevertheless, only individuals with epilepsy with a significant modulation in EEG functional connectivity from T1 to T0 experienced a significant clinical benefit of cenobamate treatment regimen, regardless of the exact pharmaco-EEG interpretations of our qEEG findings. We also showed that the modulation of background EEG connectivity induced by cenobamate administration was strongly associated with cenobamate clinical response and made the most significant contribution to identifying cenobamate responders, according to regression analysis.

**Table 2:** Clinical variables and information related to epilepsy treatment of each subject enrolled.

Sex	Age	Etiology	EEG focus	VNS	VNS response	Age of onset	Epilepsy duration	ASMs T0 (mg/day)	CNB dose (mg/day)	ASMs T1 (mg/day)	CNB response
M	45	Structural	F right	YES	NO	1	44	OXC 3600, ZNS 700, PB 200	200	OXC 2700, ZNS 700, PB 200	NO
F	58	Structural	Mul	YES	YES	2	56	PER 12, LCS 400, CLN 1.4	100	PER 12, LCS 400, CLN 1.4	YES
F	56	Unknown	T left	YES	NO	12	44	ZNS 400, LCS 500	200	ZNS 400, LCS 300	NO
M	54	Structural	T left	NO	–	41	13	CBZ 800, ZNS 300	200	CBZ 600, ZNS 300	NO
M	30	Genetic	Mul	YES	YES	1	29	FBM 2400, OXC 1800, ZNS 500	300	FBM 2400, OXC 1200, ZNS 500	YES
M	60	Structural	FT left	NO	–	49	11	BRV 100, CBZ 700, LCS 300, LMT 400, FB 100, CLB 10	50	BRV 100, CBZ 700, LCS 300, LMT 400, FB 100, CLB 10	NO
M	40	Unknown	FT left	NO	–	4	36	VPA 1800, LCS 400, BRV 200, PB 100	100	VPA 1800, LCS 400, BRV 200, PB 100	YES
F	58	Unknown	T bil	YES	YES	5	53	ESL 1600, LCS 400, VPA 2000	200	ESL 1600, VPA 2000	YES
M	25	Structural	Mul	YES	YES	7	18	CBZ 2000, FB 100, VPA 1500	100	CBZ 1600, FB 100, VPA 1500	YES
M	47	Structural	T left	YES	NO	16	31	CBZ 800, TPM 700, LMT 300, CLB 20	200	CBZ 400, TPM 700, LMT 300, CLB 20	YES
F	75	Unknown	T right	NO	–	38	37	ZNS 400, LCS 400, PB 150, PER 6	100	ZNS 400, LCS 400, PB 150, PER 6	YES (SF)
F	62	Genetic	TO bil	NO	–	8	54	PER 8, CBZ 600, LEV 2000	150	PER 8, CBZ 400, LEV 2000	YES (SF)
F	29	Unknown	Mul	YES	YES	1	28	VPA 1200, PER 6	150	VPA 1200, PER 6	YES
M	16	Structural	FCT left	YES	YES	11	5	LEV 3000, LCS 400, PER 8	300	LEV 3000, LCS 200, PER 8	YES
F	65	Structural	FT left	NO	–	8	57	VPA 1600, LCS 400	200	VPA 1600, LCS 400	YES
F	32	Unknown	FT right	NO	–	8	24	ESL 1200, LMT 400, BRV 200	200	ESL 1200, BRV 200	NO

F, frontal for EEG focus, female for sex; BRV, brivaracetam; C, central; CBZ, carbamazepine acetate; CLB, clobazam; CLN, clonazepam; LCS, lacosamide; LEV, levetiracatem; LMT, lomotrigine; EEG, electroencephalogram; ESL, eslicarbazepine acetate; FBM, felbamate; M, male; Mul, Multifocal; O, occipital; PB, phenobarbital; PER, perampanel; SF, seizure free; T, temporal; TPM, topiramate; T0, before CNB introduction; T1, after 6 months from T0; VNS, vagal nerve stimulation; VPA, valproic acid; ZNS, zonisamide.

## 5 Limitations

While we have a well-selected population, the sample size is limited; thus, our results need to be replicated in a larger population. Another limitation is the short duration of follow-up, although individuals with epilepsy in our cohort had a high seizure frequency, which allowed us to evaluate clinical response to ASMs based on ILAE recommendations (2). Our sample is not perfectly homogeneous, especially due to the different sides and locations of the epileptic focus. On the one hand, this could lead to small effect sizes that are difficult to identify and isolate; on the other hand, this heterogeneity makes us unable to study in detail the regional qEEG characteristics of the epileptic focus. Moreover, the main challenge in interpreting our findings, as well as all low-resolution scalp EEG connectivity data, lies in accurately attributing the observed connectivity to a specific pathophysiological source. In the present research setting, it is difficult to distinguish between pathological (epileptic) and physiological networks. Finally, delta PLV variation was a key factor in the regression model.

## 6 Future prospects

We believe that our data provide new insights into transferring current neurophysiology research to clinical practice. Intracranial EEG and high-density EEG (≥64 channels) could provide the best experimental conditions for research purposes with their spatial resolution, but they have scarce clinical penetration due to their invasiveness, technical complexity, and costs. To overcome these technical limitations, we designed the present and previous experiments with a low-resolution (19-channel) scalp EEG. Our findings demonstrate the association between qEEG modulation and seizure frequency changes induced by cenobamate, thus suggesting the potential of qEEG metrics for epileptological clinical practice, especially as a therapeutic response biomarker (56). However, further research in this area is necessary to validate our results in larger populations and to explore additional factors that may impact treatment response in individuals with epilepsy. We believe that the integration of pharmaco-EEG analysis in epilepsy can provide insights into the clinical efficacy of ASMs, paving the way for more tailored treatment strategies for individuals with epilepsy. From a clinical perspective, the identification of accurate response biomarkers could be particularly relevant for the drug-resistant epilepsy condition. To date, drug-resistant epilepsy has no reliable biomarkers; for this reason, the ascertainment of drug-resistant epilepsy is a clinical process requiring years, thus exposing individuals with drug-resistant epilepsy to seizures and hospitalizations, as well as delaying access to specific treatments (e.g., neuromodulation and neurosurgery) that are the only chances of epilepsy resolution (57). Thus, more accurate biomarkers are desirable in this peculiar clinical scenario.

## 7 Conclusion

The present pharmaco-EEG study provides insights into the effect of cenobamate on cortical EEG activity and connectivity. Cenobamate administration can modulate the overall spectral activity and connectivity levels in individuals with drug-resistant epilepsy, and EEG changes are strongly associated with clinical response to cenobamate. Based on these findings, we suggest that connectivity analysis of pharmaco-EEG can contribute to the prediction of responsiveness to cenobamate treatment. This evidence could have remarkable clinical implications as it opens new scenarios for innovative approaches to precision medicine in individuals with epilepsy.

## Data Availability

The raw data supporting the conclusions of this article will be made available by the authors, without undue reservation.
